# Prevalence and Factors Associated With Acute Stress Disorder Among Adults Ever Infected With COVID-19 During the Ending Phase of the Pandemic in 7 Chinese Cities: Cross-Sectional Study

**DOI:** 10.2196/73002

**Published:** 2026-03-11

**Authors:** Ziying Yang, Yanqiu Yu, Hui Lu, Xu Wang, Yong Xu, Junqiang Ying, Xianying Wen, Lei Luo, Meng Wang, Muwen Liu, Xingyi Geng, Xuchong Zhao, Biyu He, Tao Liu, Remina Maimaitijiang, Jing Gu, Joseph T F Lau

**Affiliations:** 1 Department of Medical Statistics, School of Public Health, Sun Yat-sen University, No. 74, Zhongshan 2nd Road, Nonglin Street, Yuexiu District, Guangzhou, China, 86 13660017090; 2 School of Public Health, Fudan University, Shanghai, China; 3 Oujiang Laboratory (Zhejiang Lab for Regenerative Medicine, Vision, and Brain Health), Postgraduate Training Base Alliance of Wenzhou Medical University, Wenzhou, China; 4 Neijiang Center for Disease Control and Prevention, Neijiang, China; 5 Mianyang Center for Disease Control and Prevention, Mianyang, China; 6 Hangzhou Center for Disease Control and Prevention, Hangzhou, China; 7 Jinan Center for Disease Control and Prevention, Jinan, China; 8 Shanghai Municipal Center for Health Promotion, Shanghai, China; 9 Community Health Service Center of Friendship Street, Shanghai, China; 10 Department of Public Health, School of Medicine, Shihezi University, Shihezi, China; 11 Guangzhou Joint Research Center for Disease Surveillance, Early Warning, and Risk Assessment, Guangzhou, China; 12 Sun Yat-Sen University Global Health Institute, School of Public Health and Institute of State Governance, Sun Yat-Sen University, Guangzhou, China; 13 Guangdong Key Laboratory of Health Informatics, Guangzhou, China; 14 Zhejiang Provincial Clinical Research Center for Mental Disorders, The Affiliated Wenzhou Kangning Hospital, Wenzhou Medical University, 1 Shengjing Road, Lucheng District, Wenzhou, China, 86 13143882252; 15 School of Mental Health, Wenzhou Medical University, Wenzhou, China

**Keywords:** COVID-19, China, acute stress disorder, perceptions, emotions, risk factors

## Abstract

**Background:**

Acute stress disorder (ASD) among people ever infected with COVID-19 is prevalent and may lead to posttraumatic stress disorder. Soon after China relaxed their COVID-19 control measures in November 2022 or December 2022, the infection rate surged rapidly, creating huge uncertainty and stressful situations. Little is known about situations regarding ASD at the ending phase of the pandemic.

**Objective:**

The study aimed to investigate the potential of personal cognitive or emotional factors and environmental factors of ASD.

**Methods:**

A cross-sectional study was conducted among 5545 people ever infected with COVID-19 aged 18‐60 years from December 27, 2022, to January 9, 2023, living in 7 cities of China. The 5-item Chinese version of the Primary Care PTSD Screen was used to assess ASD. Multiple logistic regression analyses were performed to identify factors of ASD.

**Results:**

The prevalence of ASD was 21.2% (1174/5545). Adjusted for the background variables, significant personal risk factors (COVID-19 infection severity, cognitions including perceived high reinfection risk and perceived weak acquired natural immunity, and emotions including worry about the long-term physical harms and panic about infection of older or younger family members), and significant environmental risk factors (difficulties in getting information and medical supplies, having unvaccinated older or younger family members, and having significant others with severe COVID-19 symptoms) were identified.

**Conclusions:**

The prevalence of ASD among people ever infected with COVID-19 was noticeable. It is warranted to identify those at high risk of developing ASD and provide them with care and early interventions to prevent deterioration. Such programs may consider targeting the modifiable risk factors found in this study.

## Introduction

As of September 29, 2023, COVID-19 had caused approximately 770 million confirmed cases and over 6 million deaths globally [1]. The Chinese government responded immediately to implement the zero-COVID-19 strategy after the outbreak in Wuhan, including strict measures such as lockdown, quarantine, travel restrictions, and regular mass testing [2-4]. By November 2022, the country had recorded about 9.3 million COVID-19 cases and 29,000 related deaths [5], respectively. Subsequently, the “Twenty Measures” were announced to ease certain controls (eg, quarantine measures and testing requirements for entry to public venues). On December 7, 2022, the “New Ten Measures” removed all control measures in China [6]. Due to the previously low population immunity to COVID-19 in China and the high transmissibility of the virus, infections surged rapidly across the country [7,8]. For instance, a seroprevalence study detecting open reading frame 8 antigen reported infection rates from 61.5% (922/1500) to 80.7% (1210/1500) in Guangzhou, China, during January 5, 2023, and January 14, 2023 [9].

The pandemic has been associated with an elevated prevalence of depression in both infected and uninfected individuals [10,11]. For example, the global prevalence of depression increased by 25% during the pandemic [12]. Meta-analyses have reported depression prevalence rates from 18% to 33% in the general population [10] and 37% to 54% among people ever infected with COVID-19 during that time period [13]. People ever infected with COVID-19 may encounter stronger stressors and hence experience a higher prevalence of mental distress than their uninfected counterparts [14-20].

People ever infected with COVID-19 are at risk of developing acute stress disorder (ASD) and posttraumatic stress disorder (PTSD). ASD is a mental health disorder that can emerge shortly after a traumatic event. If the symptoms persist beyond 1 month, the diagnosis may progress to PTSD [21]. PTSD was significantly associated with depression and suicidal ideation [22,23]. Furthermore, ASD is a known predictor of PTSD [24,25] and other harms such as memory impairment [26]. Studies in China, Italy, and Singapore reported PTSD prevalence among people ever infected with COVID-19 ranging from 9.3% to 43.3% [27]. The prevalence of ASD was 25.0%, 40.0%, and 24.1% in New York City, Romania, and Spain, respectively [28-30]. In China, only 1 study has reported ASD in people ever infected with COVID-19 (71%) during the early outbreak phase [31]. The unprecedented acceleration of COVID-19 spread in the final phase of the pandemic in China created an extremely stressful social context. People ever infected with COVID-19 may worry about transmitting the virus to their family members, which can worsen their mental distress [32-34]. Given that hundreds of millions of Chinese people were infected within a few months, a substantial number were exposed to a high risk of ASD. To inform the design of prevention programs, it is crucial to understand the risk factors for ASD among people ever infected with COVID-19.

According to the diathesis-stress model and its contemporary refinements (eg, differential susceptibility), the development of mental disorders is jointly shaped by personal predispositions and contextual or environmental factors [35-38]. Based on this framework, severity of symptoms, negative cognitions, and negative emotions related to COVID-19 have been identified as personal risk factors of mental distress. For example, severe symptoms experienced during the acute phase of infection have been shown to increase the risk of long-term depression among people ever infected with COVID-19 [14]. Negative cognitions such as a perceived risk of infection were associated with negative mental health outcomes among health care workers [39,40]. Personal emotional factors commonly studied included concern about family members’ infection [41] and worry about the long-term negative effects of COVID-19 [42]. Contextual risk factors included limited access to accurate disease-related information [43], necessary supplies [44], masks [45], medicine [46], and medical care [47]. Additionally, COVID-19 infection among family members and colleagues was identified as an environmental risk factor of depression, anxiety, and insomnia among people ever infected with COVID-19 [19].

Only 5 studies were identified in our literature review that specifically investigated risk factors for ASD among people ever infected with COVID-19. A survey conducted in Pakistan (n=114) identified several risk factors of ASD, including inadequate communication, poor ward facilities, worries about family members, and financial problems [48]. A Spanish study (n=90) found risk factors, including younger age, female sex, obesity, history of psychiatric diseases, and disease severity at intensive care unit (ICU) admission [49]. Another Italian study found that hypoxemia at ICU admission was negatively associated with ASD [30]. An Indian study found that smell or taste disturbance was associated with ASD [50]. An American study found that neither demographic characteristics nor clinical characteristics (eg, ICU admission and inflammatory markers) were associated with ASD [29]. These findings indicate that a relatively narrow range of potential risk factors has been examined. To the best of our knowledge, no such ASD studies were conducted among people ever infected with COVID-19 in China, although several reported factors of ASD in populations, including both people ever infected with COVID-19 and non–people ever infected with COVID-19 [31,51-54]. Furthermore, most of such studies were conducted during the early phase of the pandemic, whereas the pandemic’s ending phase in China presented a distinct social and psychological context with its own uncertainties.

The present study investigated the prevalence of ASD and associated personal factors and contextual or environmental factors of ASD among people ever infected with COVID-19 during the ending phase of the COVID-19 pandemic (from December 2022 to January 2023) in mainland China. It is hypothesized that (1) the following personal factors will be positively associated with ASD: (a) severity of COVID-19 infection, (b) cognitive variables, including perceived risk of reinfection and perceived strong acquired natural immunity within 6 months since infection, and (c) emotional factors, including worry about long-term harms of COVID-19 infection to oneself and panic about risks of infection of one’s older or younger family members; (2) the following contextual or environmental factors will be positively associated with ASD: (a) difficulties in obtaining COVID-19 related information, (b) difficulties in obtaining drugs rapid antigen test (RAT) supplies, (c) presence of unvaccinated older or younger family members, and (d) having relatives or friends with severe COVID-19 symptoms. Most of these factors of ASD among people ever infected with COVID-19 have not been studied.

## Methods

### Participants and Data Collection

An anonymous cross-sectional study was conducted in 7 Chinese cities during December 27, 2022, and January 9, 2023. Eligible participants were adults aged 18 to 60 years who received a COVID-19 diagnosis between December 1, 2022 (when the control measures started to be loosened or removed) and the survey date. The 7 cities—Hangzhou and Shanghai (east), Guangzhou (south), Jinan (northeast), Neijiang and Mianyang (west), and Shihezi (northwest)—were purposively selected to reflect different geographic regions.

Participants were recruited using a multistage, cluster-based recruitment across the above selected cities. Administratively, Chinese cities are divided into districts, which are further subdivided into streets, and in turn, into communities. With support from local Centers for Disease Control and Prevention (CDC), at least 3 districts were selected in each city. Within each selected district, at least 1 street was randomly selected using simple random sampling from the administrative list. Within each selected street, at least 6 communities with more than 100 residents and an existing WeChat group were randomly selected from the administrative list. WeChat groups are widely used communication platforms in communities in China [55]. Centers for Disease Control and Prevention representatives or community leaders then posted the survey link to the WeChat group of each community and invited only 1 adult per household to complete the questionnaire via the Wenjuanxing platform (Changsha Ranxing Information Technology Co, Ltd) [56].

A total of 6028 completed questionnaires met the inclusion criteria. For quality control, 483 participants were excluded (completion time > 4 min: n=445; repetitive answer patterns and substantial missingness, n=38), yielding an analytic sample size of 5545.

### Ethical Considerations

The participation was voluntary and anonymous, and returning the completed questionnaire indicated informed consent. No incentives were given to the participants. Personal identifiers were not collected, and all analyses were performed on deidentified data to ensure participant privacy and confidentiality. Ethics approval was obtained from the ethics committee of the School of Public Health, Zhejiang University (ZLG202301-01).

### Measures

The overall survey comprised modules on illness perception, protective behaviors, and mental health. This study focuses on the prevalence of ASD and its associated personal and environmental factors. Findings from the other modules are reported in published papers [57].

### Background Information

Such information included the study city, community type (urban or rural area), sex, age, education level, current marital status, employment status, and chronic disease status (no chronic disease, chronic disease well controlled, and chronic disease not well controlled).

### Acute Stress Disorder Assessment

ASD was assessed by the 5-item Chinese version of the Primary Care PTSD Screen (Primary Care PTSD Screen for *Diagnostic and Statistical Manual of Mental Disorders, Fifth Revision* [PC-PTSD-5]) [58], which was also validated in a Chinese population [59]. The items have binary responses (0=no, 1=yes). Probable ASD was defined as PC-PTSD-5 total scores greater than or equal to 3 [58]. The Cronbach α was .808 in this study.

### Personal Factors

#### Severity of COVID-19 Infection

The item was as follows: “How severe were your COVID-19 symptoms?” It was rated with a 4-point Likert scale (asymptomatic, mild, severe and not hospitalized, and severe and hospitalized).

#### Cognitive Factors

The cognitive factors were as follows:

1. Perceived risk of reinfection was assessed by the item: “In the next month, there is a high possibility that I might contract or experience a reinfection of COVID-19” (“1=strongly disagree,” “2=disagree,” “3=neutral,” “4=agree,” and “5=strongly agree”). The responses were recoded into high (4 and 5), average (3), and low (1 and 2).
2. Perceived natural immunity level within the 6 months since COVID-19 infection was assessed by the item: “With COVID-19 infection, I have acquired natural immunity and would not be re-infected within the next six months” (“1=strongly disagree,” “2=disagree,” “3=neutral,” “4=agree,” and “5 =strongly agree”). The responses were recoded into strong (4 and 5), average (3), and weak (1 and 2).

#### Emotional Factors

The emotional factors were as follows:

1. Worry about long-term physical harms of COVID-19: the item was as follows: “What are the chances that the COVID-19 infection would cause serious long-term physical harms to you?” (0=no chance to 10=extremely great chances).
2. Panic about infection of older or younger family members: the item was as follows: “Do you feel panic about the high risk that older or younger family members (age >65 years and aged 3‐11 years) would contract or recontract COVID-19?” (0=no, 1=yes).

### Contextual or Environmental Factors

#### Information-Related Difficulty

Difficulties in obtaining high-quality information related to COVID-19 were assessed by the summative scores of 3 items: “Have you frequently received inconsistent or confusing COVID-19-related information from external sources?,” “Do you find it hard to determine the accuracy of the COVID-19-related information you have received?,” and “Do you feel overwhelmed and uncertain about what to do due to the rapid changes in COVID-19 related information” (0=no, 1=yes). Higher scores indicate more difficulties in obtaining high-quality information.

#### Difficulty in Obtaining Drugs or RAT Supplies

It was assessed by the item: “How difficult was it to obtain medications (eg, for fever or cough) and RAT currently?” (1=not difficult at all to 7=impossible to obtain).

#### Vaccination and Infection Status of Family Members, Relatives, or Friends

The vaccination and infection status were as follows:

1. Having unvaccinated family members aged greater than or equal to 65 years: the item was as follows: “Do you have any unvaccinated family members aged greater than or equal to 65 years?” (0=no, 1=yes).
2. Having unvaccinated family members aged 3‐11 years: the item asked: “Do you have any unvaccinated family members aged 3‐11 years?” (0=no, 1=yes).
3. Having relatives or friends having severe COVID-19 symptoms: the item was as follows: “Do you know of any relatives or friends having developed severe COVID-19 symptoms?” A 4-point Likert scale was used (none, only a few, some, and many).

### Statistical Analysis

The distributions of studied variables are presented. Univariate logistic regression models were used to test the associations between the background factors and ASD; univariate odds ratio and 95% CIs were estimated. Multivariable logistic regression models were used to identify independent correlates of probable ASD. For each of the considered personal and environmental factors, adjusted odds ratio (ORa) and their 95% CI were respectively estimated with adjustment for all background variables. Statistical significance was reached when the 2-tailed *P* <.05. The analyses were performed using *R* Software, version 4.2.3 (R Foundation for Statistical Computing) [60].

## Results

### Descriptive Statistics

The characteristics of the study participants are summarized in Table 1. Of the 5545 participants, the majority were female participants (n=1590, 71.3%), living in an urban area (n=4569, 82.4%), aged below 40 years (n=3422, 61.7%), had attained an undergraduate degree or above (n=3095, 55.8%), working full-time (n=4065, 73.3%), and did not have chronic diseases (n=4643, 83.7%). More than half of them had mild COVID-19 symptoms (n=2883, 52%); 0.7% (n=37) had been hospitalized. Among the participants, 25.8% (n=1433), 45.4% (n=2515), or 28.8% (n=1597) perceived a high, average, or low risk of reinfection; 21.6% (n=1197), 50.2% (n=2782), or 28.2% (n=1566) perceived strong, average, or weak natural immunity within 6 months since COVID-19 infection; 75.7% (n=4195) felt panic about infection of their older or younger family members. The majority did not have unvaccinated family members aged greater than or equal to 65 years (n=4789, 86.4%) and 3 to 11 years (n=5216, 94.1%); 52.2% (n=2897) had a few relatives or friends having severe COVID-19 symptoms. The mean scores (SD; range) of worry about long-term physical harms of COVID-19, information-related difficulty, and difficulty in obtaining drugs or RAT supplies were 6.39 (2.44; 0‐10), 1.42 (1.14; 0‐3), and 4.44 (1.30; 1‐7), respectively. The prevalence of ASD was 21.2% (n=1174; 95% CI 20.1%-22.2%).

**Table 1.** Descriptive statistics among people ever infected with COVID-19 aged 18‐60 years (N=5545).^a^

	Value
Background variables, n (%)	
City	
Guangzhou	414 (7.5)
Hangzhou	1790 (32.3)
Jinan	668 (12.0)
Mianyang	1176 (21.2)
Neijiang	1090 (19.7)
Shanghai	243 (4.3)
Shihezi	164 (3.0)
Community type	
Rural	976 (17.6)
Urban	4569 (82.4)
Sex	
Male	1590 (28.7)
Female	3955 (71.3)
Age group (y)	
18-30	1465 (26.4)
31-40	1957 (35.3)
41-50	1493 (26.9)
51-60	630 (11.4)
Education level	
Below undergraduate degree	2450 (44.2)
Undergraduate degree and above	3095 (55.8)
Current marital status	
Unmarried	1296 (23.4)
Married	4249 (76.6)
Employment status	
Not being employed	1321 (23.8)
Full-time job	4062 (73.3)
Part-time job	162 (2.9)
Chronic disease status	
None	4643 (83.7)
Yes, extremely well controlled	126 (2.3)
Yes, well controlled	564 (10.2)
Yes, not well controlled	212 (3.8)
Personal factors, n (%)	
Severity of COVID-19 infection	
Asymptomatic	65 (1.1)
Mild	2883 (52)
Severe and not hospitalized	2560 (46.2)
Severe and hospitalized	37 (0.7)
Cognitive factors, n (%)	
Perceived risk of reinfection	
Low	1597 (28.8)
Average	2515 (45.4)
High	1433 (25.8)
Perceived natural immunity level within 6 mo since COVID-19 infection	
Strong	1197 (21.6)
Average	2782 (50.2)
Weak	1566 (28.2)
Emotional factors	
Worry about long-term physical harms of COVID-19, mean (SD)	6.39 (2.44)
Panic about infection of older or younger family members, n (%)	
No	1350 (24.3)
Yes	4195 (75.7)
Contextual/environmental factors	
Information-related difficulty, mean (SD)	1.42 (1.14)
Difficulty in obtaining drugs/RAT^b^ supplies, mean (SD)	4.44 (1.3)
Having unvaccinated family members aged ≥65 years, n (%)	
No	4789 (86.4)
Yes	756 (13.6)
Having unvaccinated younger family members aged 3-11 years, n (%)	
No	5216 (94.1)
Yes	329 (5.9)
Having relatives/friends with severe COVID-19 symptoms, n (%)	
None	884 (15.9)
Only a few	2897 (52.2)
Some	1377 (24.8)
Many	387 (7.0)
Dependent variable, n (%)	
Acute stress disorder	
No	4371 (78.8)
Yes	1174 (21.2)

a Chronic disease includes any of hypertension, diabetes, chronic lung disease, myocardial infarction, heart failure, cerebrovascular disease, dementia, and ulcerative diseases such as gastric ulcer, liver disease, and tumor. Range of worry for long-term impact of COVID-19, information difficulty, and difficulty in obtaining drugs or rapid antigen test supplies are 0‐10, 0‐3, and 1‐7, respectively. Acute stress disorder is defined using a cutoff of 3 or higher.

b RAT: rapid antigen test.

### Factors of ASD

#### Background Factors

Univariate logistic regression analyses showed that living in Neijiang (vs Guangzhou), female sex, ages greater than 30 years (vs 18‐30 y), being currently married, having a part-time job (vs not being employed), and having chronic diseases (both well controlled and not well controlled vs no chronic disease) were more likely than others to develop ASD. Conversely, university or above education and full-time employment (vs not being employed) were negatively associated with ASD (Table 2).

**Table 2.** The association between background factors and acute stress disorder among people ever infected with COVID-19 aged 18‐60 years (N=5545).

	ORu^a^ (95% CI)
City	
Guangzhou	Ref^b^
Hangzhou	1.11 (0.84-1.46)
Jinan	1.29 (0.95-1.75)
Mianyang	1.15 (0.87-1.53)
Neijiang	1.45 (1.09-1.93)^c^
Shanghai	1.43 (0.97-2.09)
Shihezi	0.65 (0.39-1.10)
Community type	
Rural	Ref
Urban	0.97 (0.82-1.15)
Sex	
Male	Ref
Female	1.41 (1.21-1.63)^d^
Age group (y)	
18‐30	Ref
31‐40	1.24 (1.05-1.48)^e^
41‐50	1.25 (1.05-1.50)^e^
51‐60	1.38 (1.10-1.73)^e^
Education level	
Below undergraduate degree-	Ref
Undergraduate degree and above	0.85 (0.74-0.96)^c^
Current marital status	
Unmarried	Ref
Married	1.28 (1.09-1.50)
Employment status	
Not being employed	Ref
Full-time job	0.81 (0.70-0.94)
Part-time job	1.49 (1.04-2.12)^c^
Chronic disease status^f^	
None	Ref
Yes, extremely well controlled	1.08 (0.70-1.67)
Yes, well controlled	1.48 (1.21-1.81)^d^
Yes, not well controlled	3.71 (2.81-4.91)^d^

a ORu: univariate odds ratio.

b Ref: reference group.

c *P*<.05.

d *P*<.001.

e *P*<.01.

f Chronic disease includes any of hypertension, diabetes, chronic lung disease, myocardial infarction, heart failure, cerebrovascular disease, dementia, and ulcerative diseases such as gastric ulcer, liver disease, and tumor.

#### Personal Factors

The results of the adjusted logistic regression analyses are shown in Table 3. Regarding the personal factors, having severe symptoms that did not hospitalize (ORa=5.56, 95% CI 2.00-15.47; reference group: asymptomatic) and a condition that hospitalized (ORa=9.74, 95% CI 2.88-32.93; reference group: asymptomatic) were both positively associated with ASD. Regarding the cognitive factors, both higher perceived risk of reinfection (average vs low: ORa=1.68, 95% CI 1.40-2.02; high versus low: ORa=3.62, 95% CI 3.00-4.38) and lower perceived natural immunity level within 6 months since infection (weak vs strong: ORa=1.51, 95% CI 1.25-1.82) were positively associated with ASD. In terms of emotional factors, higher levels of worry about long-term physical harms of COVID-19 (ORa=1.47, 95% CI 1.42-1.52) and panic about risk of infection of older or younger family members (ORa=2.00, 95% CI 1.68-2.39) were positively associated with ASD.

**Table 3.** Logistic regression on personal/environmental factors and acute stress disorder among people ever infected with COVID-19 aged 18‐60 y (N=5545). The models were adjusted for city, urban/rural area, sex, age group, education level, current marital status, employment status, and chronic disease status.

	ORa^a^ (95% CI)
Personal factors	
Severity of COVID-19 infection	
Asymptomatic	Ref^b^
Mild	2.48 (0.89-6.90)
Severe and not hospitalized	5.56 (2.00-15.47)^c^
Severe and hospitalized	9.74 (2.88-32.93)^d^
Cognitive factors	
Perceived risk of reinfection	
Low	Ref
Average	1.68 (1.40-2.02)^d^
High	3.62 (3.00-4.38)^d^
Perceived natural immunity level within 6 months since COVID-19 infection	
Strong	Ref
Average	1.13 (0.95-1.35)
Weak	1.51 (1.25-1.82)^d^
Emotional factors	
Worry about long-term physical harms of COVID-19	1.47 (1.42-1.52)^d^
Panic about infection of older or younger family members	
No	Ref
Yes	2.00 (1.68-2.39)^d^
Contextual/environmental factors	
Information-related difficulty	1.42 (1.34-1.51)^d^
Difficulty in obtaining drugs/RAT^e^ supplies	1.35 (1.28-1.44)^d^
Having unvaccinated family members aged ≥65 years	
No	Ref
Yes	1.48 (1.23-1.78)^d^
Having unvaccinated family members aged 3-11 years	
No	Ref
Yes	1.35 (1.04-1.75)^f^
Having relatives/friends with severe COVID-19 symptoms?	
None	Ref
Only a few	1.65 (1.29, 2.10)^d^
Some	3.99 (3.10, 5.13)^d^
Many	6.95 (5.13, 9.41)^d^

a ORa: adjusted odds ratio.

b Ref: reference group.

c *P*<.01.

d *P*<.001.

e RAT: rapid antigen test.

f *P*<.05.

#### Contextual or Environmental Factors

Significant contextual or environmental factors included higher levels of information-related difficulty (ORa=1.42, 95% CI 1.34-1.51), difficulty in obtaining drugs or RAT supplies (ORa=1.35, 95% CI 1.28-1.44), having unvaccinated family members aged greater than or equal to 65 years (ORa=1.48, 95% CI 1.23-1.78), having unvaccinated family member aged 3‐11 years (ORa=1.35, 95% CI 1.04-1.75), and having relatives or friends having severe COVID-19 symptoms (only a few versus none: ORa=1.65, 95% CI 1.29-2.10; some versus none: ORa=3.99, 95% CI 3.10-5.13; many versus none: ORa=6.95, 95% CI 5.13-9.41).

## Discussion

Substantially, about one-fifth of the sampled people ever infected with COVID-19 showed ASD, especially among those who were female, older, less educated, married, not in full-time employment, and having chronic disease conditions. Supporting the initial hypotheses, significant personal risk factors of ASD (severity of infection, perceived high reinfection risk, perceived low natural immunity level postinfection, worry about long-term physical harms of COVID-19, and panic about infection of older or younger family members) and contextual or environmental risk factors of ASD (difficulties in getting information and medical supplies, the unvaccinated status of older and younger family members, and having relatives or friends with severe COVID-19 symptoms) were identified.

The ASD prevalence in our study was lower than the rate of 71% reported in another Chinese study [31] and that of some other countries [28-30]. Such discrepancy could be attributed to variations in the stages of the COVID-19 pandemic as the other studies were conducted during the early phases. The early outbreak was characterized by fear of a novel and more severe virus, coupled with the experience of strict lockdowns—both of which are established population-level stressors [10,13,61]. In contrast, our study was conducted during the ending phase, which was dominated by the milder Omicron variant and followed the lifting of all restrictive measures. Given that disease severity is a key risk factor for ASD [14], the population-level shift toward less severe clinical manifestations, together with the removal of lockdown-related stressors, provides a parsimonious explanation for the observed difference in ASD prevalence.

Among the personal factors, the severity of COVID-19 infection was positively associated with ASD, consistent with previous research [14]. This relationship can be understood through several pathways. First, the direct physiological impact of a severe infection, including high fever, intense bodily pain, and dyspnea, can be perceived as a life-threatening physical trauma, which constitutes a core diagnostic criterion for stress-related disorders [62-65]. Second, the management of severe symptoms often necessitates intensive efforts, including seeking urgent medical care and securing necessary supplies, thereby amplifying situational stressors and potentially exacerbating the perception of threat [62,64,65]. Importantly, the markedly elevated ASD risk observed in the “severe and hospitalized” group likely extends beyond a simple gradient of symptom intensity. During the study period in China, the decision to hospitalize a patient was shaped by a complex interplay of clinical necessity, health care resource availability, and local pandemic policies [66,67]. Therefore, hospitalization itself constituted a distinct and potent psychosocial stressor, characterized by forced isolation from one’s daily milieu, reliance on an overburdened emergency health care system, anxieties related to treatment costs, and direct confrontation with the potential for severe health outcomes [68,69]. These factors—stemming from health care system interaction and institutionalization—may operate independently of, or synergistically with, the physiological distress of the infection to jointly exacerbate acute stress responses. Consequently, our findings highlight that individuals who experienced more severe COVID-19 illness, particularly those who required hospitalization, constitute a critical high-risk subgroup warranting prioritized screening and early psychological intervention to mitigate the potential progression to PTSD.

Corroborating previous studies among health care workers, a higher perceived risk of reinfection was positively associated with ASD [39,40]. This empirical relationship underscores the significant role of threat appraisal in the development of stress-related disorders. Within the framework of fear appeal theories, our results highlight the public health imperative of calibrating risk communication: excessively elevated perceptions of susceptibility may precipitate maladaptive emotional outcomes such as ASD [70,71], whereas unrealistically low perceptions can undermine protective behaviors [72]. In the context of an evolving pathogen like SARS-CoV-2, this underscores the critical public health imperative to disseminate timely and accurate information. For instance, during Omicron-predominant waves, public communication should concurrently reinforce the high transmissibility (to motivate booster vaccination) and the substantially reduced severity (to alleviate excessive fear and prevent ASD), thereby fostering both protection and psychological resilience. Conversely, a stronger perceived level of natural immunity 6 months post-infection served as a protective factor against ASD, likely mediated by a reduction in perceived susceptibility. However, the durability of infection-induced immunity is finite, waning over time [73,74], and public awareness regarding its extent and duration is often incomplete or inaccurate [75]. This knowledge gap underscores the necessity for clear communication that not only delineates the properties and limitations of natural immunity but also proactively promotes booster vaccination and the superior, more robust protection conferred by hybrid immunity [76].

We postulate that confidence in vaccine-induced immunity may buffer against the anxiety stemming from waning natural immunity. Given the demonstrated efficacy of social media in shaping pandemic-related perceptions [77], these platforms represent an indispensable channel for the prompt and accessible dissemination of evolving epidemiological and clinical evidence to the public.

Persistent worry is a recognized precursor to anxiety [78] and ASD [79]. In line with this, our study identified a significant positive association between worry about the long-term physical harms of COVID-19 and ASD, a finding consistent with prior research [41,42]. The prevalence of such concerns is understandable given evidence on long COVID; a meta-analysis reported a pooled prevalence of 43% [80], encompassing symptoms such as fatigue, cognitive impairment, and cardiovascular sequelae [81,82]. As the majority of the Chinese population was infected during our survey period, long COVID emerged as a widespread public health concern, thereby elevating the population-level risk for ASD—a dynamic potentially applicable to other global populations. The persistence of these concerns may be partly attributable to limited public understanding of the long-term effects of COVID-19 [83]. Therefore, evidence-based information on the prevalence and severity of long COVID is warranted to mitigate excessive worry. Furthermore, our results indicate that panic about the risk of infection for vulnerable family members (the older adults and children) was independently associated with ASD, echoing its link to depression [19]. The increased odds of ASD among participants with unvaccinated older or younger family members substantiates this concern, as unvaccinated infected older adults face a significantly elevated risk of severe outcomes and mortality [84]. This underscores the continued importance of prompting vaccination in these vulnerable demographics. Collectively, these findings highlight the central role of emotional disturbance in ASD. Consequently, fostering adaptive emotional regulation should be a cornerstone of secondary prevention strategies for ASD. Maladaptive regulation strategies, such as rumination and catastrophizing, were robustly linked to mental distress during the pandemic [85]. Evidence-based brief interventions, including cognitive behavioral therapy [86] and mindfulness-based programs, which have demonstrated efficacy in ameliorating maladaptive emotional regulation [87], should be integrated into such preventive frameworks.

Regarding environmental factors, both difficulties in accessing timely and consistent COVID-19 information and in obtaining essential medical supplies were significantly associated with ASD. Information-related difficulties (eg, inconsistent messaging and information overload) may generate profound uncertainty and hinder effective coping, thereby elevating the risk of anxiety and ASD [53,88]. Furthermore, the significant association between difficulties in obtaining drug or RAT supplies and ASD underscores a critical and tangible dimension of the pandemic’s psychological impact. Unlike informational uncertainty, the scarcity of essential medical supplies posed a direct threat to personal and family health, leaving individuals feeling vulnerable and powerless to manage their illness [89,90]. These findings collectively demonstrate that ensuring both informational clarity and physical access to key medical resources is critical for public mental health protection. Future epidemic responses must therefore prioritize robust supply chains and equitable distribution systems to mitigate these potential stressors.

Similarly, other studies have found that limited access to accurate COVID-19–related information was associated with mental health problems [43]. To our knowledge, the relationship between difficulty in accessing related information and ASD specifically has been underreported. The lack of clear information generates uncertainty or misconceptions, which can increase anxiety and hence the risk of ASD [91]. This risk was exacerbated during the unprecedented surge in infections, which created an urgent public demand for updated, authoritative information regarding transmission dynamics, disease severity, access to health care, and government policies [92-94]. As supported by previous findings, infected individuals with limited understanding of the situation can experience significant uncertainty and panic [93]. Therefore, ensuring public access to regular, accurate, and authoritative information from official sources is a critical public health strategy to mitigate ASD during crises. This also necessitates active efforts to combat the spread of misinformation on social media [95].

Our study further identified that having relatives or friends who experienced severe COVID-19 symptoms was positively associated with ASD among people ever infected with COVID-19. This aligns with existing evidence linking such exposure to mental distress [96]. This association may be explained by several mechanisms. First, emotional contagion [97] could play a role, whereby the psychological distress of close contacts adversely affects the participant’s own mental state. This suggests that people ever infected with COVID-19 should be counseled on the potential mental health impact their illness disclosures may have on significant others. Second, providing emotional and instrumental support to infected significant others may impose a caregiver burden on participants [98], contributing to their distress. This burden can be particularly prolonged and severe when significant others develop long COVID, necessitating ongoing monitoring for ASD not only in the patients themselves but also in their caregivers [99]. Community-based programs that foster mutual aid networks could help alleviate this burden. In addition, integrating interventions designed to bolster psychological resources such as resilience and optimism—which are known to buffer against stress [100]—into support services for people ever infected with COVID-19 could represent an effective secondary prevention strategy.

As a universal public health strategy, it is recommended to raise awareness among stakeholders regarding the signs and consequences and preventive measures of ASD or PTSD, including the use of evidence-based brief positive psychology interventions [101]. The establishment of a comprehensive, one-stop digital platform for ASD screening and support—integrating self-assessment tools, online or printable self-help materials, emotional support hotlines, and facilitated referral pathways—could be highly beneficial. Individuals screening positive for ASD should be proactively monitored for the potential development of PTSD in the subsequent months. Furthermore, the risk factors identified in this study could be operationalized into a structured checklist to facilitate the provision of personalized interventions. The development of algorithm-driven digital tools to select specific brief interventions from a pre-established library, based on an individual’s unique risk profile, represents a promising avenue for scalable and efficient mental health support. Proactive development of such systems is crucial, as public health capacity is often strained during pandemics, underscoring the importance of preparedness during interpandemic periods.

The present study has interesting findings but also several limitations. First, our multistage, cluster-based recruitment across purposively selected cities, and voluntary, online participation means the sample is not strictly probability based; response metrics or weights were unavailable, so generalizability should be interpreted with caution, and WeChat-based recruitment may have introduced coverage and self-selection bias. Second, as this is a cross-sectional study, causal relationships cannot be inferred. Third, this study did not assess several important factors, including interpersonal factors (eg, perceived social support [52]) and baseline dispositional traits (eg, emotional stability and emotion-regulation skills [102,103]), which are known predictors of ASD. Additionally, potential confounders such as a history of previous COVID-19 infection were not measured. Fourth, the variable “having unvaccinated family members aged greater than or equal to 65” measured a composite household stressor that conflates 2 concerns. Future research would benefit from measuring these 2 factors—family age structure and vaccination status within the household—separately to elucidate their unique and interactive contributions to mental distress. At last, we used logistic regression for risk identification, which does not assess mediation or moderation. Structural equation modeling or related methods based on multi-item scales are needed to clarify pathways and interconnections in future work.

To summarize, this study found noticeable ASD prevalence among people ever infected with COVID-19 at the explosive ending phase of the COVID-19 pandemic in China and identified some significant personal and environmental factors. The findings are implicative. First, the risk of ASD and PTSD is noticeable. Preparations for universal prevention, screening, brief secondary intervention, stocking of key materials, information campaign, and follow-ups are greatly warranted to prevent ASD and PTSD. Second, health promotion may consider modifying the identified risk factors of ASD. Longitudinal studies are required in future pandemics to better understand the relationship between ASD and PTSD. It is also important to investigate the level of PTSD during the postpandemic stage. The findings may improve prevention of ASD and PTSD in future pandemics.

## Abbreviations

ASD: acute stress disorder
ICU: intensive care unit
ORa: adjusted odds ratio
PC-PTSD-5: Primary Care PTSD Screen for *Diagnostic and Statistical Manual of Mental Disorders, Fifth Revision*
PTSD: posttraumatic stress disorder
RAT: rapid antigen test
